# Obituary

**Published:** 1916-05

**Authors:** 


A Memorial service was held by the Buffalo Historical Society, April 18, in honor of the late Dr. Roswell Park and the late Dr. Ernest Wende. Hon. Adelbert Moot presented the memorial to Dr. Wende; Dr. Chas. G. Stockton and Chancellor Charles P. Norton to Dr. Park, the latter dwelling especially on Dr. Park's connection with the University of Buffalo.

